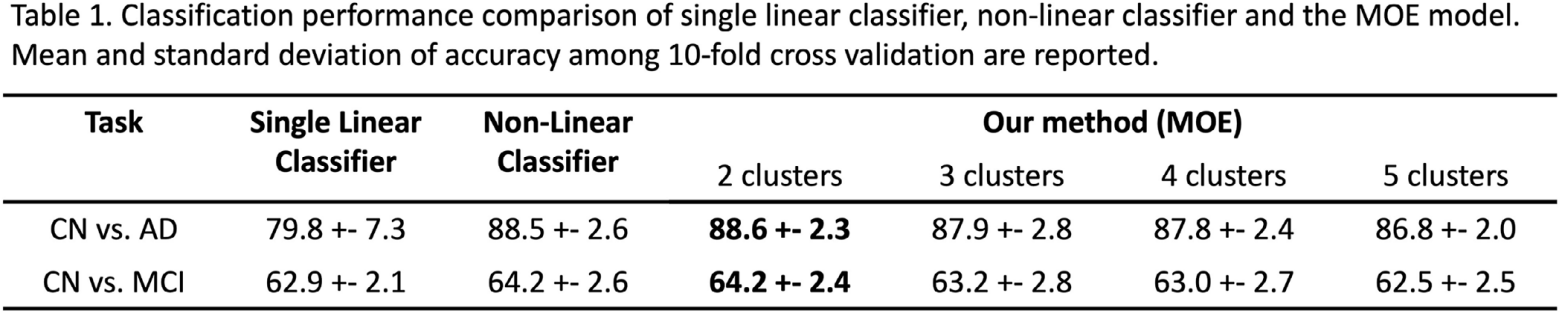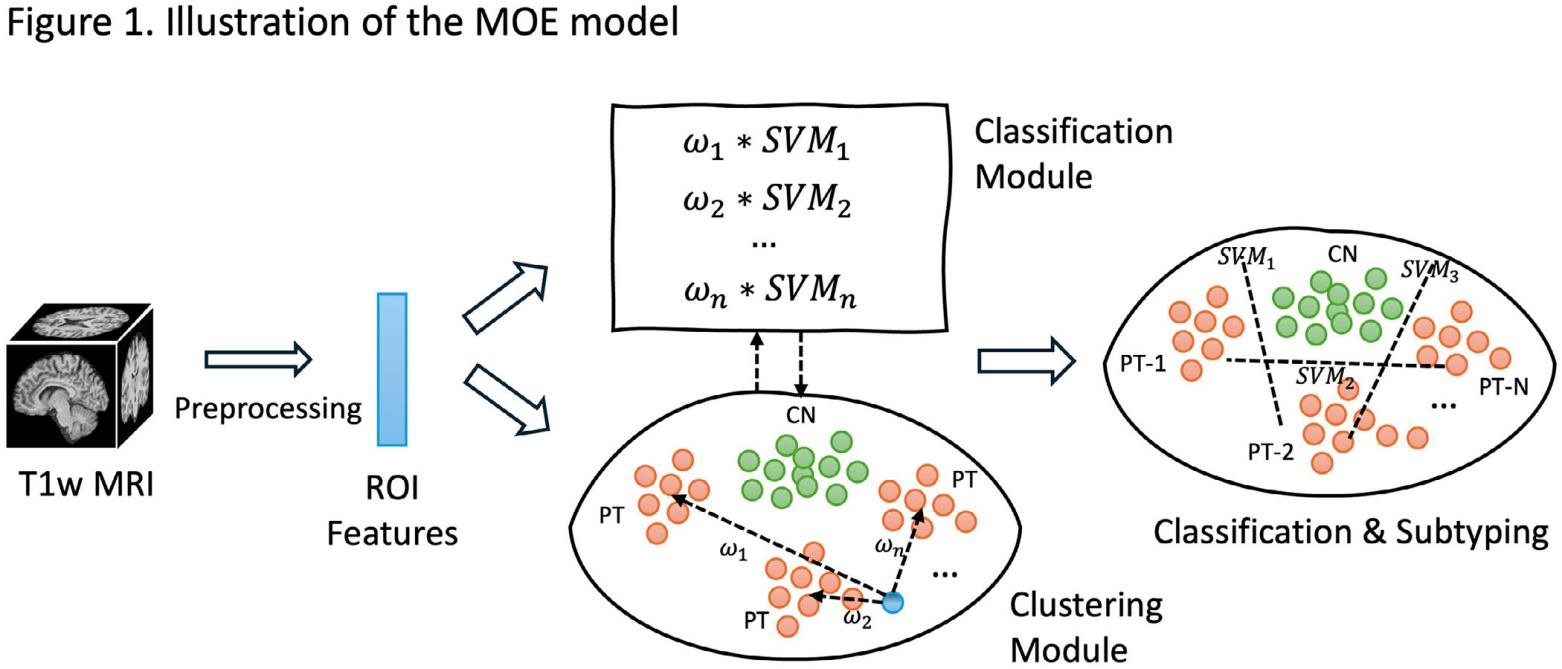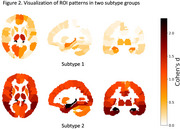# Characterizing heterogeneous atrophy patterns of Alzheimer's disease dementia with mixture‐of‐experts (MOE) for joint classification and clustering

**DOI:** 10.1002/alz70856_103682

**Published:** 2025-12-26

**Authors:** Yuanwang Zhang, Yong Fan

**Affiliations:** ^1^ University of Pennsylvania, Philadelphia, PA, USA; ^2^ Perelman School of Medicine, University of Pennsylvania, Philadelphia, PA, USA

## Abstract

**Background:**

Neurodegeneration diseases are heterogeneous, with individuals presenting varying patterns of disease progression. This inherent heterogeneity poses a major challenge for accurate diagnosis and treatment. Identifying biological meaningful subtypes among affected individuals is critical for advancing the understanding of disease mechanisms and improving diagnostic precision. This study introduces a simultaneous clustering and classification method to 1) classify subjects as either cognitively unimpaired (CU) or Alzheimer's disease (AD) and 2) group AD individuals into distinct subtypes.

**Method:**

This study includes 2,374 participants with baseline T1‐weighted images obtained from the Alzheimer's Disease Neuroimaging Initiative (ADNI). Images are processed using the t1‐volume pipeline of the Clinica toolbox, yielding 120 Region‐of‐Interest (ROI) values, representing the average gray matter density values of anatomical regions defined by AAL2 template. These ROI values are analyzed within a mixture‐of‐experts (MOE) framework guided by a clustering module. Each expert is a linear classifier and a non‐linear decision boundary is obtained through a weighted combination of these classifiers with the weights learned jointly by the classifiers in a discriminative manner and the clustering module to group individuals based on their feature similarity. This framework is implemented in an end‐to‐end training paradigm.

**Result:**

The classification performance was assessed using 10‐fold Stratified‐KFold validation, with mean accuracy and standard deviation reported. The MOE model demonstrated the best performance when the number of clusters was set to two, outperforming a single SVM classifier and achieving performance comparable to a non‐linear classifier. The MOE model also identified two subtypes among AD subjects, as illustrated in Figure 2. Both subtypes exhibited significant hippocampal atrophy, while subtype 2 showing more severe atrophy than the another, which is consistent with late‐stage AD pathology.

**Conclusion:**

This study demonstrated the effectiveness of MOE model in both classification and characterization of the AD heterogeneity. By approximating non‐linear decision boundaries, the MOE model enhances the classification performance while simultaneously providing interpretable subtype information. This method offers valuable insights into the automatic and interpretable diagnosis of Alzheimer's disease and potentially other neurodegenerative diseases.